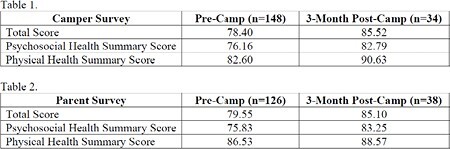# 71 Burn Camp Outcomes: Capturing Changes in Pediatric Quality of Life

**DOI:** 10.1093/jbcr/irae036.063

**Published:** 2024-04-17

**Authors:** Kerry Mikolaj, Brad Jackson, Nichole Schiffer, Trudy J Boulter, Genevieve Kierulf

**Affiliations:** Children's Hospital Colorado, Aurora, CO; Children's Hospital Colorado, Denver, CO; Children's Hospital Colorado, Aurora, CO; Children's Hospital Colorado, Denver, CO; Children's Hospital Colorado, Aurora, CO; Children's Hospital Colorado, Denver, CO; Children's Hospital Colorado, Aurora, CO; Children's Hospital Colorado, Denver, CO; Children's Hospital Colorado, Aurora, CO; Children's Hospital Colorado, Denver, CO

## Abstract

**Introduction:**

The Pediatric Quality of Life Inventory (PedsQL) is an outcome measure used to assess health related quality of life in children and adolescents. This tool is used in healthy, acute, and chronic health conditions to assess physical and psychosocial functioning. We applied this measure with our pediatric burn survivors and their caregivers. Within a burn camp setting, we evaluate progress towards programming goals relating to physical functioning, emotion regulation, social skill challenges, and academic participation. In order to further assess the impact of burn camp experiences on quality of life, we are in the process of collecting PedsQL data from patients in the burn clinic who have not attended burn camp.

**Methods:**

PedsQL surveys were sent to campers ages 8-18 years old and their caregivers prior to burn camp and again 3 months post-camp from 2018-2023. Surveys consisted of questions relating to physical, emotional, social, and school functioning. Survey data were coded and average scores for each functioning scale were used to generate a total score, psychosocial health score, and physical health score for each subset of surveys.

**Results:**

PedsQL scores increased following burn camp among both camper and caregiver reports for all outcomes measured. High scores are indicative of a better quality of life rating (see Tables 1 & 2).

**Conclusions:**

PedsQL surveys were trialed as a measure of burn camp outcomes from 2018-2019, showing positive changes among quality of life measures post-camp for both campers and caregivers. Since 2019 we have significantly expanded our data set, showing similar trends, and now plan to compare PedsQL scores of campers with those of burn injured children seen in our burn clinic, who did not attend camp.

**Applicability of Research to Practice:**

The PedsQL is a validated outcome measure used to assess quality of life in children with a wide range of medical conditions. Our research supports the use of this tool in evaluating the success of medical specialty camps.